# Shaping of Signal Transmission at the Photoreceptor Synapse by EAAT2 Glutamate Transporters

**DOI:** 10.1523/ENEURO.0339-16.2017

**Published:** 2017-06-12

**Authors:** Stephanie Niklaus, Lucia Cadetti, Colette M. vom Berg-Maurer, André Lehnherr, Adriana L. Hotz, Ian C. Forster, Matthias Gesemann, Stephan C.F. Neuhauss

**Affiliations:** 1 Institute of Molecular Life Sciences, University of Zurich, Zurich, CH-8057, Switzerland; 2 Institute of Physiology, Zurich, CH-8057, Switzerland; 3 Life Science Zürich Graduate Program – Neuroscience, Zurich, CH-8057, Switzerland

**Keywords:** Excitatory amino acid transporter, glutamate, retina, zebrafish

## Abstract

Photoreceptor ribbon synapses tonically release glutamate. To ensure efficient signal transmission and prevent glutamate toxicity, a highly efficient glutamate removal system provided by members of the SLC1 gene family is required. By using a combination of biophysical and *in vivo* studies, we elucidate the role of excitatory amino acid transporter 2 (EAAT2) proteins in synaptic glutamate homeostasis at the zebrafish photoreceptor synapse. The main glutamate sink is provided by the glial EAAT2a, reflected by reduced electroretinographic responses in EAAT2a-depleted larvae. EAAT2b is located on the tips of cone pedicles and contributes little to glutamate reuptake. However, this transporter displays both a large chloride conductance and leak current, being important in stabilizing the cone resting potential. This work demonstrates not only how proteins originating from the same gene family can complement each other’s expression profiles and biophysical properties, but also how presynaptic and glial transporters are coordinated to ensure efficient synaptic transmission at glutamatergic synapses of the central nervous system.

## Visual Abstract

**Figure:**
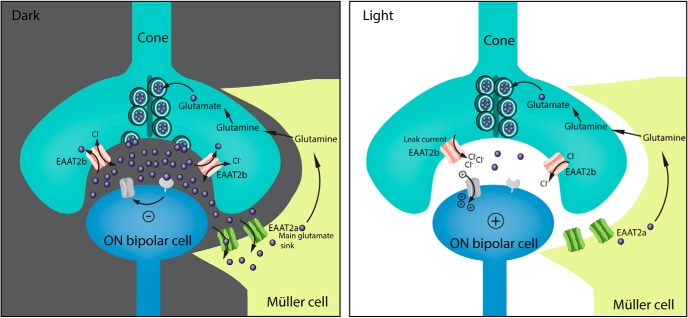

## Significance Statement

Glutamate transporters are key regulators of glutamate homeostasis. Here we analyze two players of glutamate homeostasis at the zebrafish photoreceptor synapse. This report demonstrates how paralogous glutamate transporters emerging from a whole-genome duplication event acquired a complementary expression pattern and adopted different biophysical characteristics that allow modulation of the synapse and signal transmission in a specialized manner.

## Introduction

Excitatory amino acid transporters (EAATs) are high-affinity glutamate transporters that regulate the extracellular concentration of glutamate in the vertebrate brain and retina. The transport of glutamate is an electrogenic process driven by the cotransport of three sodium and one hydrogen and the countertransport of one potassium ion (Zerangue and Kavanaugh, 1996). In addition, a thermodynamically uncoupled chloride conductance, whose magnitude differs between EAATs, is associated with the glutamate transport. This chloride conductance can modulate the cell’s membrane potential (Picaud et al., 1995; Palmer et al., 2003; Veruki et al., 2006; Wersinger et al., 2006). Photoreceptors possess highly specialized ribbon synapses, where vesicles are tethered to provide tonic release of glutamate (Morgans, 2000; Schmitz, 2009). In contrast to nonribbon synapses, where the depolarization of the presynaptic terminal induces the release of a few vesicles of neurotransmitter, cone ribbon synapses allow the simultaneous release of hundreds of vesicles. This can result in exceptionally high glutamate concentrations within the synapse, making efficient glutamate uptake capacities essential to ensure an optimal signal-to-noise ratio, precisely terminate a signal, and prevent neurotoxic glutamate concentrations (Morgans, 2000). As a consequence, EAATs are highly expressed in the retina. In mammals, the main retinal glutamate uptake is conducted by Müller glia cells through EAAT1 (GLAST; White and Neal, 1976; Harada et al., 1998; Rauen et al., 1998; Barnett and Pow, 2000; Rauen, 2000; Levinger et al., 2012; Tse et al., 2014). However, in most other vertebrates including zebrafish (*Danio rerio*), the glutamate transporter expressed on Müller glia cells is EAAT2 (GLT-1; Eliasof et al., 1998a, b). Interestingly, EAATs are also present at photoreceptor terminals, where glutamate uptake is accompanied by a subtle regulation of the presynaptic potential (Eliasof and Werblin, 1993; Vandenbranden et al., 1996; Gaal et al., 1998; Roska et al., 1998; Hasegawa et al., 2006; Rowan et al., 2010; Szmajda and Devries, 2011).

In the present study, we focus on the role of EAAT2 at the photoreceptor synapse of the cone-dominant zebrafish retina. The zebrafish genome harbors two *eaat2* genes that originated from a whole-genome duplication event ∼350 million years ago (Gesemann et al., 2010b). For EAAT2, we found an unusual case of both spatial and functional subfunctionalization (division of the ancestral gene function among the two paralogs, as reviewed by Glasauer and Neuhauss [2014]). Biophysical properties of the transporters differ in dependence of their biological requirements. EAAT2a is expressed in Müller glia cells, and knockdown of this glial transporter significantly reduces the electroretinogram (ERG) b-wave amplitude, indicating that EAAT2a removes the main load of synaptic glutamate. EAAT2b is located presynaptically in cone pedicles. Loss of EAAT2b does not significantly reduce the ERG b-wave, suggesting that it interferes only slightly with the removal of cleft glutamate: its action could be limited to the cone presynaptic terminal in agreement with its proposed function of accelerating the transient cone response (Rowan et al., 2010). Furthermore, with its high chloride conductance and the presence of a significant leak current, EAAT2b may stabilize the dark resting potential of cones. This presents an intriguing case of divergent evolution of an ancestral protein leading to subfunctionalized proteins at the photoreceptor synapse, supporting vision through different mechanisms.

## Methods

### Fish maintenance

Zebrafish *(Danio rerio)* of the Tübingen and Wik strain were kept in a 14-h/10-h light/dark cycle under standard conditions as previously described (Mullins et al., 1994). Larval stages were raised in E3 embryo medium (5 mm NaCl, 0.17 mm KCl, 0.33 mm CaCl_2_, 0.33 mm MgSO_4,_ 10^−5^% methylene blue) at 28°C. All experiments were performed in accordance with the ARVO Statement for the Use of Animals in Ophthalmic and Vision Research and were approved by the local authorities (Veterinäramt Zürich TV4206).

### *In situ* hybridization

Cloning of the *eaat2* genes into the TOPO pCRII vector (TA Cloning Kit Dual Promoter, Invitrogen) is described elsewhere (Gesemann et al., 2010a). Plasmids containing the genes were linearized for SP6 and T7 *in vitro* transcription and purified with phenol-chloroform. Digoxigenin (DIG)-labeled antisense RNA probes were generated using DIG-RNA-labeling kit (Roche Diagnostics). Larval whole-mount and adult retina *in situ* hybridization was done on 5-d postfertilization (5-dpf) larvae and adult retinal cross sections. Detailed protocol of *in situ* hybridization is described by Huang et al. (2012). Briefly, the tissue was treated with proteinase K and postfixed with 4% paraformaldehyde (PFA) before prehybridization at 64°C. Hybridization of RNA probes was done at 64°C overnight. On day 2, after several stringency washes at 64°C, probes were blocked in 1× Roche blocking solution in Tris/NaCl/Tween. Anti-DIG AP antibody was applied overnight at 4°C. On day 3, after several washing steps, signal was detected by incubation in staining buffer. Stained embryos/retinal sections were fixed with PFA and imaged in glycerol (whole-mount) with an Olympus BX61 light microscope. Images were processed and assembled using Adobe Photoshop and Adobe Illustrator CS5.

### Generation of antibodies

Chickens were immunized using the peptide H_2_N-CKL KEN LGE GLE NDE V-CONH_2_ to raise chicken anti-EAAT2a antibodies. Antibodies against EAAT2b were raised in guinea pigs using the peptides H_2_N-CKL KAN LGE GKK NDE V-CONH_2_ and H_2_N-CKG AAK YVI KKS LQF KS-CONH_2_. Antibodies were raised in a 87-d classic program and affinity-purified against the corresponding peptides by Eurogentec (Seraing, Belgium).

### Immunohistochemistry

Larvae (5 dpf) or adult eyes were fixed in 4% PFA in PBS, pH 7.4, or 2% trichloroacetic acid for 30 min at room temperature. Samples were cryoprotected in 30% sucrose in PBS overnight at 4°C and embedded in cryomatrix (Tissue Tek OCT Compound, Sakura Finetek) using liquid N_2_ to immediately freeze the samples. Sections of 16–18 μm were mounted onto Superfrost slides (Thermo Fisher Scientific). Slides were air-dried at room temperature and stored at –20°C. Before use, slides were thawed at 37°C for 30 min and washed in PBS, pH 7.4, for 10 min. Blocking solution (10% normal goat serum, 1% bovine serum albumin, 0.3% Tween 20 in PBS, pH 7.4) was applied for at least 45 min at room temperature, and primary antibodies diluted in blocking solution were incubated overnight at 4°C. The following antibodies were used: mouse anti–glutamine synthetase (EMD Millipore, MAB302) 1:700, mouse anti–Zpr-1 (Fret43, a commonly used marker labeling red-green double cones; Larison and Bremiller, 1990; Zebrafish International Resource Center) 1:400, chicken anti-EAAT2a 1:100, and guinea pig anti-EAAT2b 1:100. The immunoreaction was then detected using fluorescently labeled secondary antibodies (goat anti-mouse Alexa Fluor 488 or 568, goat anti–guinea pig Alexa Fluor 488 or 568, all from Invitrogen, and rabbit anti-chicken IgY Cy5 from Jackson ImmunoResearch or rabbit anti-chicken Alexa Fluor 488 from Invitrogen) diluted 1:1000 in PBS. Bodipy TR Methyl Ester (Invitrogen) was used to counterstain green fluorescence. It was applied 1:300 in PDT (PBS with 1% DMSO and 0.1% Triton) for 20 min after washing the secondary antibodies.

Slides were coverslipped and imaged with a SP5 and a TCS LSI confocal microscope (both Leica Microsystems). Images were then processed with Imaris (Bitplane) and postprocessed using Gimp imaging processing software, Adobe Photoshop, and Adobe Illustrator CS5.

### Histology

Whole larvae were fixed in 4% PFA overnight at 4°C. Larvae were dehydrated in series of increasing ethanol concentrations in PBS (50%, 70%, 80%, 90%, 95%, and 100% ethanol). After dehydration, larvae were incubated in a 1:1 ethanol Technovit 7100 (Heraeus Kulzer) solution (1% Hardener 1 in Technovit 7100 basic solution) for 1 h followed by incubation in 100% Technovit solution overnight at room temperature. Larvae were then embedded in plastic molds in Technovit 7100 polymerization medium and dried at 37°C for 1 h. 3-μm-thick sections were prepared with a microtome, mounted onto slides, and dried at 60°C. Sections were stained with Richardson–Romeis (0.5% borax, 0.5% Azur II, 0.5% methylene blue), and slides were mounted with Entellan (Merck). Images were taken in the bright-field mode of a BX61 microscope (Olympus).

### Gene knockdown

Two different EAAT2a translation-blocking morpholinos were used for injections into the one-cell stage of the zebrafish embryo. Titration injections were performed to find the optimal dose with highest knockdown efficiency but no toxic side effects. For EAAT2a morpholino 1, CATCATCCACAACTGTCAGGCTGGC (position –22 to –03), the injected amount was 1.3 ng. For EAAT2a morpholino 2, CGTGCTTCGGCATCATCCACAACTG (position –12 to –13), two different amounts were injected, 1.8 and 3.6 ng (referred to as low and high dose, respectively). EAAT2b morpholino 1, GATCTCCACTTGCTTCTGCATCTTC (position –04 to –21), was injected at a dose of 1.8 ng, and morpholino 2, GAGTTTCACAACAGTTTGCTAGACA (position –65 to –41), was injected at a dose of 9 ng.

Before dilution, stock morpholinos were heated to 65°C for 5 min and subsequently diluted with nuclease free H_2_O to the desired concentration (injection mix contained 0.04% phenol red for color indication). The injection amount was 1 nl. The standard control morpholino was injected into sibling embryos. Injected embryos were raised in E3 containing methylene blue at 28°C until 5 dpf.

Efficiency of knockdown was quantified by measuring the fluorescence of antibody staining on wild-type (WT) and morphant retinal sections (imaged with the same confocal microscope settings). For EAAT2a, a region of interest (ROI) reaching from photoreceptors to basal processes of Müller cells was selected to measure the fluorescence. For EAAT2b, the ROI was placed over an area of the outer plexiform layer (OPL). Because EAAT2b staining is present only in OPL, background fluorescence [ROI set on inner nuclear layer (INL)] was subtracted from fluorescence in OPL. Two ROIs were set per animal. One-way ANOVA with Tukey *post hoc* test was performed to statistically analyze the difference in fluorescence.

### Assessment of retinal morphology in WT and EAAT2 knockdown animals

Retinal morphology of EAAT2a and EAAT2b morphant and WT animals was analyzed on cryosections, and several parameters were statistically compared. 5-dpf WT (*n* = 13) and larvae injected with EAAT2a MO 1 (1.3 ng; *n* = 13), EAAT2a MO 2 (*n* = 12), EAAT2b MO 1 (1.8 ng; *n* = 13), and EAAT2b MO 2 (9 ng; *n* = 13) were fixed, embedded in cryomatrix, and sectioned at 16 µm before immunofluorescent staining with Zpr-1 (EAAT2b morphants) and glutamine synthetase (EAAT2a morphants; see Materials and Methods, “Immunohistochemistry”). The retinal thickness was measured on confocal images using Fiji software. Retinal thickness was assessed on three different locations per larva. The average of the three measurements was used for statistical analysis. The length of Müller cells (in EAAT2a morphants) as well as the overall cone length (EAAT2b morphants) was assessed the same way. In total, three cones and three Müller cells per larvae were analyzed (same sample sizes as for retinal thickness analysis), and the average of the three measurements was used for statistical analysis. One-way ANOVA followed by Tukey *post hoc* test was performed using SPSS.

### Electroretinography

ERG recordings were performed on 5-d-old morphant larvae of either sex and their control injected siblings as previously described by Makhankov et al. (2004) with some minor adaptations. Larvae were dark-adapted for at least 30 min, and all preparations before the actual recording were done under a red light, preventing bleaching of photoreceptors. The larval eye was removed from the eyecup and placed onto filter paper on top of a 1.5% agarose gel in E3, in which the reference electrode was inserted. The recording electrode (micropipette filled with E3) was placed onto the center of the cornea. 100-ms light stimuli of five different intensities (log-4 to log0) were given with an interstimulus interval of 7 s. The light intensity of log0 corresponds to 6800 lux or 75 W/m^2^.

b-wave amplitudes and the time from light onset to the b-wave peak were compared using mixed repeated-measures ANOVA followed by Tukey and Games–Howell *post hoc* test. Results are shown in a box-and-whisker plot (b-wave amplitudes and time from light onset to peak), where the bottom and top of the box represent the first and third quartile, respectively, with the median represented as a line within the box and whiskers reaching to minimum and maximum values obtained.

### Oocyte experiments

*Xenopus* oocytes were purchased from Ecocyte Bioscience or were obtained from the Institute of Physiology, University of Zurich. The oocytes were kept at 16°C for 2–5 d. Capped cRNA was microinjected into *Xenopus* oocytes (40–80 ng per oocyte), and membrane currents were recorded 2–4 d later.

With the exception of the chloride-free solution, the extracellular recording solution comprised (in mm) 96 NaCl, 2 KCl, 1.8 CaCl_2_, 1 MgCl_2_, and 5 HEPES, pH 7.5. l-Glutamate was obtained from Sigma; DL-TBOA (dl-threo-β-benzyloxyaspartic acid) was obtained from Tocris. For chloride-free solutions, all chloride salts were replaced with gluconate salts. To increase the uncoupled current, the oocytes were dialyzed in chloride-free solution for 24 h, and the recordings were then performed in a solution in which chloride was substituted with the more permeant ion SCN^-^. The approximated chloride equilibrium potential, E_Cl-_, for the injected oocytes was estimated by calculating the reversal potential of I_Cl(Ca)_ induced by 4-Br A23187 (Sigma), a calcium ionophore, as described by Wadiche et al. (1995).

Radiolabeled glutamate uptake was performed under voltage clamp at –60 mV (or –15 mV when uptake at the chloride equilibrium potential was investigated). Currents were recorded during application of 100 μm [^3^H]l-glutamate for 100 s. The specific activity of the [^3^H]l-Glutamate in this solution was 20 Ci/mol. For control, we used the same protocol on uninjected oocytes. After application of [^3^H]l-glutamate, oocytes were first washed in Ringer solution and then lysed in a scintillation vial containing 2% SDS. Radioactivity was later measured. Two-electrode voltage-clamp recordings were performed using a Turbo Tex-03x amplifier (NPI), and signals were acquired with a Digidata 1440A (Molecular Devices). Recording electrodes with resistance <1 MΩ contained 3 m KCl. Currents were acquired at 10 kHz and low-pass filtered at 20 or 500 Hz in dependence of the recording protocol.

Data analysis was performed using Axon pClamp10 and GraphPad Prism software. To determine *K_m_*, dose–response curves were fitted with the Michaelis–Menten relationship included with the GraphPad Prism software, *Y* = *I*_max_ × *X*/(*K_m_* + *X*), where *I*_max_ is the maximum current in the same units as *Y* and *K_m_* is the Michaelis–Menten constant, in the same unit as *X*.

## Results

### EAAT2 paralogs are complementarily expressed in the retina

**Figure 1.**
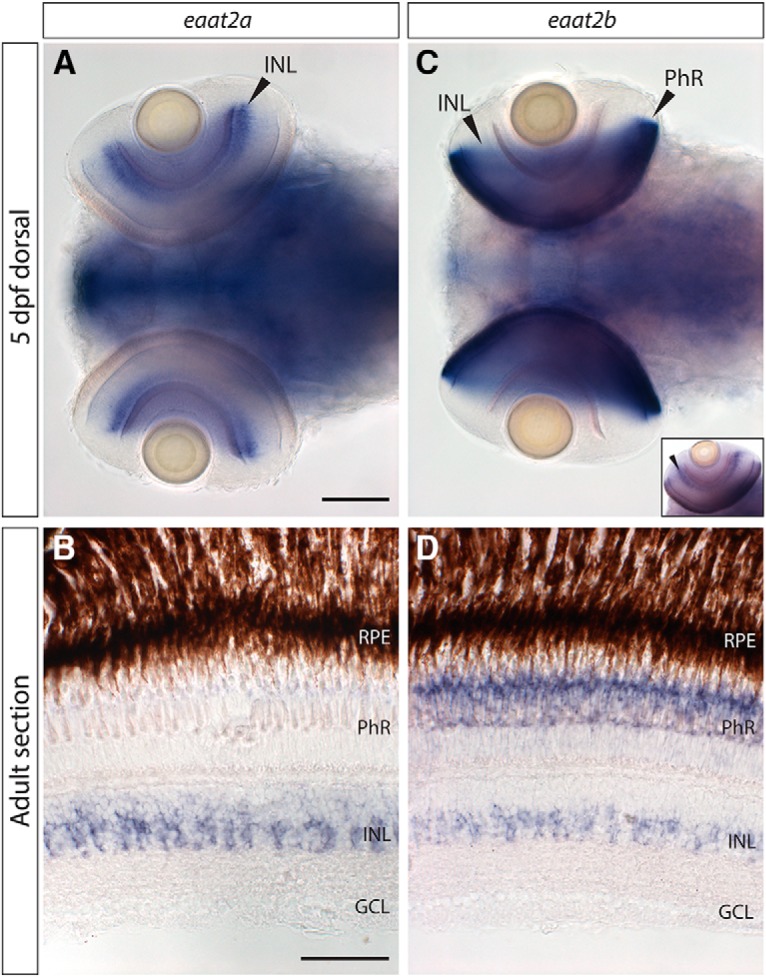
Transcript expression of excitatory amino acid transporter 2 (*eaat2*) paralogs. ***A***, ***B***, *eaat2a* mRNA is strongly expressed in the inner nuclear layer (INL) in the retina, in both 5-d postfertilization (5-dpf) larvae (***A***) and adult retina (***B***). Additionally, extremely low transcript levels can be found in photoreceptors (***B***). ***C***, ***D***, mRNA of *eaat2b* is expressed in photoreceptors and weakly in the INL throughout different developmental stages (***C***, in 5-dpf larvae; ***D***, in adult retinal sections). Small inset in ***C*** shows *eaat2b in situ* staining in an eye of a whole-mount larva that has been only shortly stained, to better visualize expression in the INL. Scale bar in ***A*** is 100 µm; also applies to ***C***. Scale bar in ***B*** corresponds to 50 µm; also applies to ***D***.

A phylogenetic analysis of excitatory amino acid transporter genes revealed that the zebrafish genome harbors two *eaat2* (also called *glt-1* or *slc1a2*) genes (Gesemann et al., 2010a). Analyzing the transcript expression of the two transporters in the zebrafish retina, we found strong *eaat2a* expression in the INL in close proximity to the inner plexiform layer (IPL) and additionally very weak expression in photoreceptors (Fig. 1*A*, *B*). In contrast, *eaat2b* mRNA was present in the photoreceptor cell layer and in low concentration in the INL (Fig. 1*C*, *D*). Staining in the photoreceptor layer was present throughout all cone photoreceptors; however, the layer containing nuclei of rods was unstained, suggesting *eaat2b* to be cone specific.

**Figure 2.**
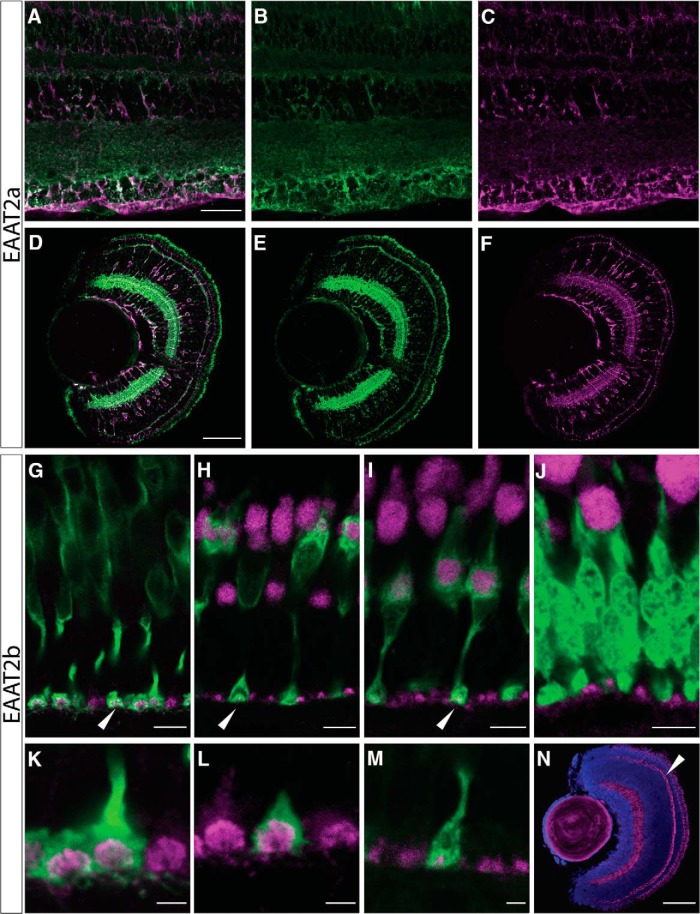
Protein expression of EAAT2 paralogs. ***A–F***, Double immunostaining of EAAT2a (green) and glutamine synthetase (magenta) in adult (***A***) and larval (5 dpf; ***D***) retinal sections confirm expression of EAAT2a in Müller glia cells. Separated channels are shown in ***B*** (adult), ***E*** (5 dpf; EAAT2a, green channel only), ***C*** (adult), and ***F*** (5 dpf; glutamine synthetase, magenta channel only). Scale bar in ***A*** is 30 µm; also applies to ***B*** and ***C***. Scale bar in ***D*** is 50 µm; also applies to ***E*** and ***F***. ***G–N***, EAAT2b protein is expressed in a dotted manner in the outer plexiform layer (OPL) in all cone pedicles, but it is not expressed in rods. EAAT2b antibody staining (magenta) on adult retinal sections stained with Zpr-1 (red-green double cones, ***G***) and on retinal sections of zebrafish expressing GFP in blue cones (***H***), UV cones (***I***), and rods (***J***) confirms that EAAT2b is cone specific and is spared from rod spherules. ***K–M*** show zoom-ins of the cone pedicles expressing EAAT2b (magenta) in red-green double cones (***K***), blue cones (***L***), and UV cones (***M***). ***N*** shows larval (5 dpf) expression of EAAT2b in magenta together with a nuclear counterstain (DAPI, blue). Scale bars in ***G–J*** are 7 µm. Scale bars in ***K–M*** are 2 µm. Scale bar in ***N*** is 30 µm.

To confirm cellular identity and obtain information about subcellular distribution of the protein, we generated paralog-specific peptide antibodies. Consistent with the *in situ* hybridization data, immunostainings against EAAT2a on larval and adult retinal sections revealed expression of EAAT2a in Müller glia cell membranes (Fig. 2*A–F*). EAAT2a seems to be specific to Müller cells, as no immunofluorescent signal could be detected in photoreceptors. The staining intensity within Müller glia cells appeared increased in the OPL and IPL, where glutamatergic synapses are tightly ensheathed by Müller glia cells. The nature of the labeled cells was confirmed by double-labeling with the glial marker glutamine synthetase, which colocalizes with EAAT2a (Fig. 2*A*, *D*). Consistent with the mRNA distribution, we found dotted antibody staining against EAAT2b protein in the OPL (Fig. 2*G–N*). The appearance of the staining indicated localization of EAAT2b in cone pedicles, whereas rod spherules were devoid of EAAT2b protein. To further characterize the photoreceptor subtype-specific expression, we used transgenic fish lines expressing GFP in different photoreceptor cell types [Tg(zfSWS1–5.5A:EGFP), Tg(zfSWS2–3.5A:EGFP), Tg(zfRh1-3:EGFP); Hamaoka et al., 2002; Takechi et al., 2003, 2008]. EAAT2b is expressed in the presynaptic terminal of all cone subtypes (UV-, blue-, red-, and green-sensitive cones; Fig. 2*G–I*, *K–M*); however, consistent with the *in situ* hybridization results, rod terminals do not express this transporter (Fig. 2*J*). Remarkably, no EAAT2b immunofluorescence could be observed in cells of the INL, suggesting that local protein concentrations in the membranes of these cells are rather low, preventing immunostaining.

### EAAT2 paralogs differentially shape signal transmission in the cone synapse

**Figure 3.**
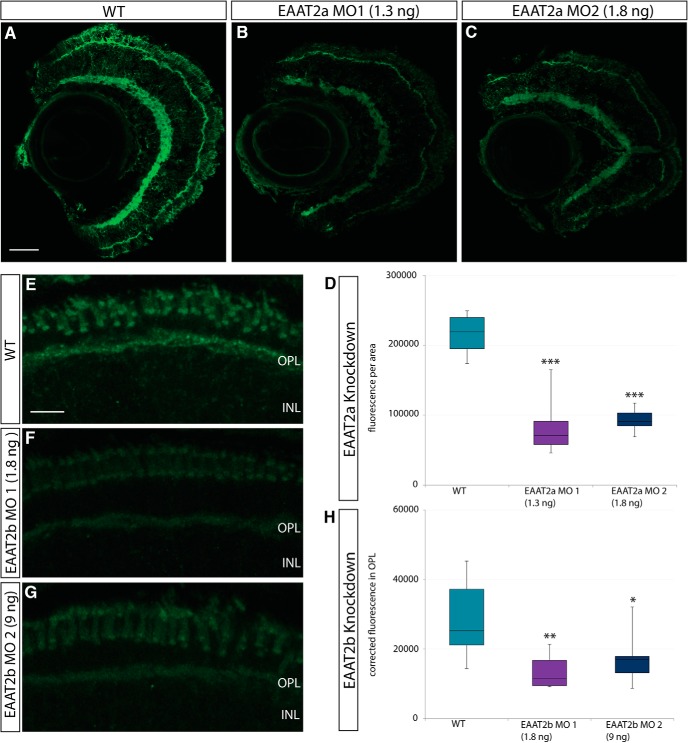
Confirmation of knockdown. ***A–C***, Immunostaining of EAAT2a on WT (***A***) and EAAT2a morphant [***B***, 1.3 ng EAAT2a morpholino (MO) 1; ***C***, 1.8 ng EAAT2a MO 2] retinal sections (5 dpf). ***D***, Box-and-whisker plot of analysis of fluorescence of WT, EAAT2a MO 1, and EAAT2a MO 2 injected animals stained with anti-EAAT2a antibody. Statistical analysis reveals a highly significant (*p* < 0.001) reduction of fluorescence for both MOs. ***E–G***, Retinal sections of WT (***E***) and EAAT2b morphant (***F***, 1.8 ng EAAT2b MO 1; ***G***, 9 ng EAAT2b MO 2) larvae stained with anti-EAAT2b antibody. ***H***, Fluorescence was measured in the OPL, and background fluorescence (taken from area in INL) was subtracted. Fluorescence of WT and morphant immunostaining is plotted in a box-and-whisker plot and shows a significant (*p* < 0.01) and slightly significant (*p* < 0.05) decrease in fluorescence in animals injected with 1.8 ng EAAT2b MO 1 and 9 ng EAAT2b MO 2, respectively. EAAT2a WT, *n* = 6; EAAT2a MO 1, *n* = 8; EAAT2a MO 2, *n* = 8; EAAT2b WT, *n* = 10; EAAT2b MO 1, *n* = 10; EAAT2b MO 2, *n* = 10. Scale bar in ***A*** is 30 μm; also applies to ***B*** and ***C***. Scale bar in ***E*** is 10 μm; also applies to ***F*** and ***G***.

To characterize the function of EAAT2 transporters in zebrafish vision, we generated knockdown larvae using different morpholino antisense oligonucleotides. Quantification of fluorescence intensities in morphant and WT eyes demonstrated the efficiency of protein knockdown in 5-dpf larvae (Fig. 3).

**Figure 4.**
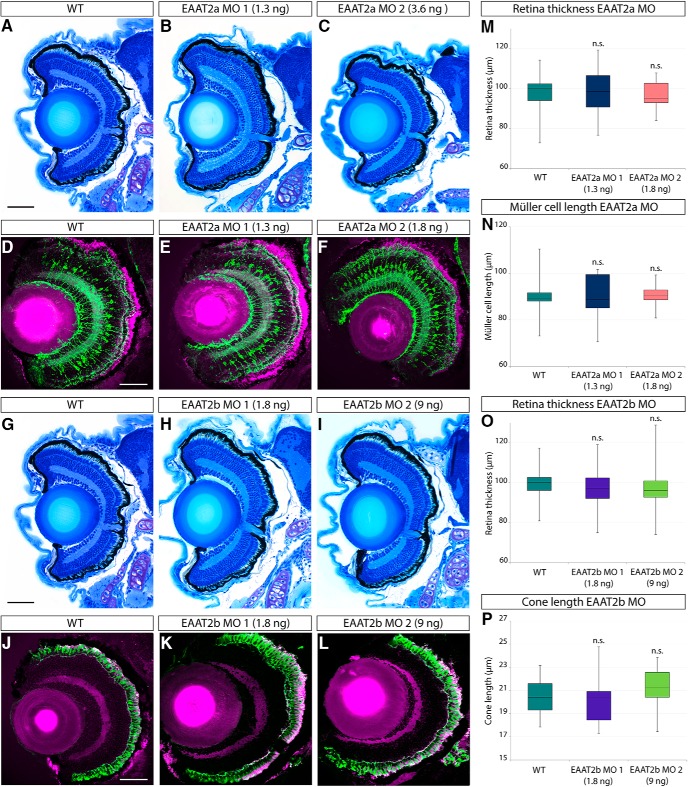
Retinal histology of EAAT2 morphant zebrafish larvae. Histologic analysis of retinal sections of WT, EAAT2a, and EAAT2b morphant zebrafish larvae (5 dpf) stained with Richardson–Romeis (***A–C*** and ***G–I***). Immunostaining of glutamine synthetase (green) labeling Müller glia cells counterstained with Bodipy (magenta; ***D–F***) of WT and EAAT2a morphant (***E***, EAAT2a MO 1; ***F***, EAAT2a MO 2) 5-dpf retinal sections. Anti–Zpr-1 immunostaining (labeling red and green cones, shown in green) on WT (***J***) and EAAT2b morphant (***K***, EAAT2b MO 1; ***L***, EAAT2b MO 2) retinal sections counterstained with Bodipy (magenta). Knockdown of neither EAAT2a (***B***, EAAT2a MO 1; ***C***, EAAT2a MO 2) nor EAAT2b (***H***, EAAT2b MO 1; ***I***, EAAT2b MO 2) causes any defect in retinal lamination. Thickness of the retina was assessed on WT and morphant larvae and did not reveal any significant difference in the thickness of the retina in either EAAT2a or EAAT2b morphants (***M***, ***O***), yielding *p* values of 0.997 and 0.935 for EAAT2a MO 1 and EAAT2a MO 2, respectively, and 0.658 and 0.922 for EAAT2b MO 1 and EAAT2b MO 2 (all in comparison to WT). Moreover, knockdown of EAAT2a does not significantly influence Müller glia cell length (***N***), nor does the loss of EAAT2b result in cone length alteration (***P***). Statistical analysis of the cell length yielded *p* values of 0.969 and 0.989 for EAAT2a MO 1 and EAAT2a MO 2, respectively, and 0.911 and 0.631 for EAAT2b MO 1 and EAAT2b MO 2 (in comparison to WT). All scale bars are 50 µm. Scale bar in ***A*** also applies to ***B*** and ***C***; scale bar in ***D*** also applies to ***E*** and ***F***; scale bar in ***G*** also applies to ***H*** and ***I***; and scale bar in ***J*** also applies to ***K*** and ***L***.

Both EAAT2a- and EAAT2b-deficient animals showed no apparent overall morphologic abnormalities (Fig. 4*A–C*, *G–I*) or abnormalities in the cell shape of Müller glia cells (Fig. 4*D–F*) or red and green cones (Fig. 4*J–L*). Statistical analysis confirmed this assessment, as no significant difference in retinal thickness between WT and EAAT2a (Fig. 4*M*) or EAAT2b morphant larvae (Fig. 4*O*) could be detected. Furthermore, neither the length of Müller cells (Fig. 4*N*) nor that of cones (Fig. 4*P*) was influenced by knockdown of EAAT2a and EAAT2b, respectively, indicating that the knockdown does not impair retinal development. However, EAAT2a morphant larvae displayed behavioral changes (no quantifications done) and abnormal body bends, similar to the ones described in *tnt* mutants that carry a point mutation in *eaat2a* (McKeown et al., 2012).

To analyze the functional role of EAAT2 in the zebrafish retina, we performed ERG on 5-d-old morphant larvae. The ERG measures the sum field potential generated by retinal cells on light exposure. We used the amplitude of the b-wave at varying light intensities as a functional readout of outer retinal function. The b-wave reflects the activation of ON bipolar cells after photoreceptor hyperpolarization after a light stimulus. In a typical WT ERG, the large b-wave masks the a-wave, resulting in no or only a tiny a-wave visible. At 5 d, rods are not yet functionally integrated into the larval retina; therefore, the measured light responses are exclusively cone driven (Branchek, 1984; Branchek and Bremiller, 1984). To analyze the ERG kinetics, the time from light stimulus onset to the peak of the b-wave was compared between control and morphant animals.

**Figure 5.**
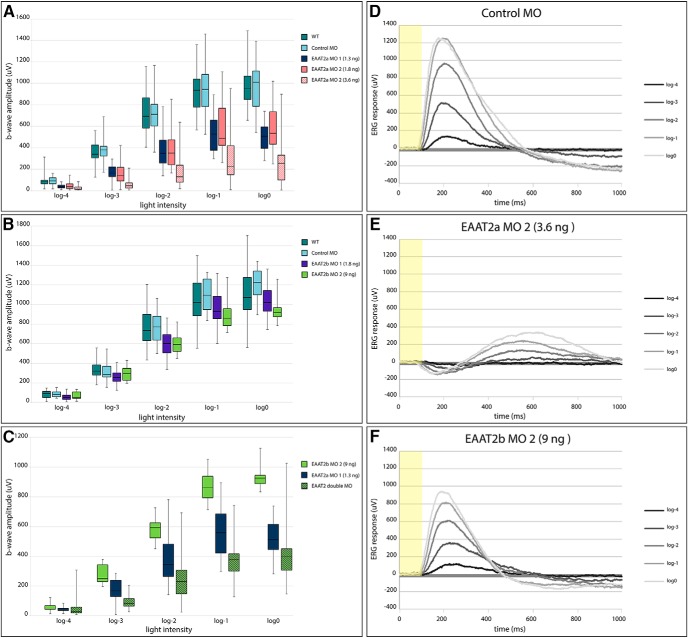
Box-and-whisker plots of ERG b-wave amplitudes of EAAT2a and EAAT2b morphant zebrafish larvae and representative ERG traces. ***A***, Knockdown of EAAT2a results in a highly significant (*p* < 0.001) reduction of the ERG b-wave amplitude in comparison to both WT and control injected animals throughout all light intensities (log-4 to log0). For EAAT2a MO 2, we could demonstrate a dose dependence, resulting in a highly significant difference (*p* < 0.001) between the low dose (1.8 ng) and the high dose (3.6 ng) for the bright light intensities (log-1 and log0) and a significant difference (*p* < 0.01) for the medium light intensities (log-3 and log-2). WT, *n* = 33; control MO, *n* = 25; EAAT2a MO 1, *n* = 23; 1.8 ng EAAT2a MO 2, *n* = 27; 3.6 ng EAAT2a MO 2, *n* = 24. ***B***, Knockdown of EAAT2b only mildly interferes with the ERG b-wave. There is an overall tendency in EAAT2b-depleted animals to have a slightly reduced ERG b-wave amplitude. This results in a slight statistical significance (*p* < 0.05) between WT and EAAT2b morphant (MO 1) at log-4 and a highly significant (*p* < 0.001) reduction at log-3 and log-2. Further, there is a slightly significant (*p* < 0.05) reduction between EAAT2b morphants (MO 1) and control morphants for log-2. When using EAAT2b MO 2, we obtained a significant (*p* < 0.01) reduction of the ERG b-wave amplitude in comparison to control morphants at log0 and in comparison to WT at log-2. WT, *n* = 39; control MO, *n* = 11; EAAT2b MO 1, *n* = 39; EAAT2b MO 2, *n* = 16. ***C***, The function of EAAT2b could be demonstrated by double knockdown of both EAAT2 paralogs. Under such conditions, when glutamate uptake by Müller glia cells was impaired, we could show an even further reduction of the ERG b-wave amplitude in the double morphants in comparison to EAAT2a morphant larvae [slightly significant (*p* < 0.05) at log0, significant (*p* < 0.01) at log-1, and highly significant (*p* < 0.001) at log-3 and log-2]. EAAT2a MO 1, *n* = 23; EAAT2b MO 2, *n* = 16; double MO, *n* = 32. ***D–F***, Representative ERG traces of control MO injected larvae (***D***), EAAT2a morphant (***E***, high dose of MO 2), and EAAT2b morphant (MO 2) larvae (***F***). Yellow bar represents light stimulus (starting at time 0, ending at 100 ms).

Loss of EAAT2a leads to a reduction of the ON response. EAAT2a-deficient larvae showed a highly significant reduction in b-wave amplitude throughout all different light intensities tested, indicating that this glial transporter is fundamental for uptake of the synaptic glutamate (Fig. 5*A*). b-wave amplitudes of larvae injected with 1.3 ng morpholino 1 or low-dose morpholino 2 were diminished by >40% throughout all different light intensity flashes. b-wave amplitudes of larvae injected with high-dose morpholino 2 showed a reduction of 75% or more in comparison to WT and control injected larvae. Furthermore, we show a clear dose dependence of morpholino 2, leading to significant differences in the b-wave amplitude between low- and high-dose EAAT2a morphants. This was observed for all light intensities except the lowest intensity stimulus (log-4). However, even at high doses of injected EAAT2a morpholino, we could still observe a small ERG b-wave, indicating that there is either no complete protein knockdown or that different glutamate transporters present at the first visual synapse take over a comparable function.

In contrast to EAAT2a, knockdown of EAAT2b resulted in only a mild ERG phenotype. The medians of ERG b-wave amplitudes of EAAT2b morphant larvae were slightly reduced compared with WT and control morphant ERG, but were statistically significant only for some light intensities (see legend, Fig. 5*B*).

To artificially augment glutamate concentrations in the cleft and disrupt the glutamate–glutamine cycle between photoreceptors and Müller glia cells, double injections of 1.3 ng EAAT2a and 9 ng EAAT2b morpholino were performed. Interestingly, except for dim light conditions, b-wave amplitudes of double-knockdown larvae were significantly lower than those of EAAT2a morphants (Fig. 5*C*). This nicely demonstrates the function of EAAT2b by illustrating that glutamate clearing capacity has further been neutralized in the EAAT2 double morphants.

**Figure 6.**
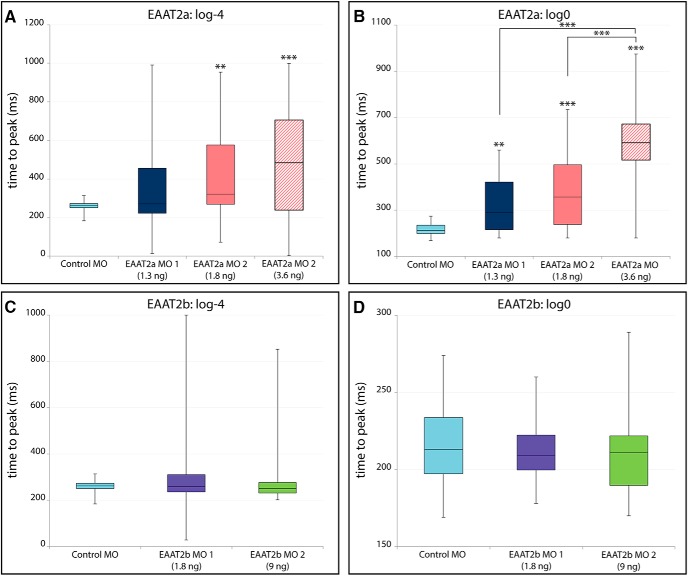
Time-to-peak analysis of ERG recordings. Box-and-whisker plots of time (ms) from onset of light stimulus (0 ms) to the peak of the b-wave. EAAT2a morphant larvae display changed ERG kinetics. At low light levels (***A***), low and high doses of EAAT2a MO 2 result in significant (*p* < 0.01) and highly significant (*p* < 0.001) increases in the time to peak, respectively. At bright light levels (***B***), EAAT2a morphant (MO 1) larvae show a significant (*p* < 0.01) increase in the time to peak, whereas both levels of MO 2 result in a highly significant (*p* < 0.001) increase in the time to peak. ERG response of EAAT2b morphant fish was not decelerated in dim light conditions (***C***) or in bright light (***D***).

Knockdown of EAAT2a affected not only the b-wave amplitude, but also its kinetics. In general, the ERG response in EAAT2a-depleted animals was slower (representative ERG traces shown in Fig. 5*D*) than in WT or control animals (Fig. 5*E*). The time to peak at low light intensity stimuli (log-4) was significantly increased in EAAT2a-depleted larvae. Also at bright light (log0), the ERG response was still decelerated, resulting in a significant increase in the time to peak in EAAT2a-depleted animals (Fig. 6*A*, *B*). Because ON bipolar cell activity is decreased and delayed in EAAT2a morphants, the b-wave no longer dominates the a-wave. Therefore, the a-wave (electronegative wave preceding the positive b-wave) becomes apparent in an EAAT2a morphant ERG (Fig. 5*E*). ERG kinetics in EAAT2b-depleted larvae is unaffected (Fig. 6*C*, *D*; representative ERG traces are shown in Fig. 5*F*).

### Biophysical properties of EAAT2 paralogs

Glutamate uptake by EAAT proteins is electrogenic, being associated with the movement of charges through the cell membrane: three Na^+^ ions and one proton enter the cell for each transport cycle, and one K^+^ ion moves out (Zerangue and Kavanaugh, 1996; Levy et al., 1998; Owe et al., 2006). In addition, on binding of glutamate and sodium, a thermodynamically uncoupled chloride current is associated with these transporters (Fairman et al., 1995; Wadiche and Kavanaugh, 1998). These intrinsic electrogenic properties of glutamate transporters allow evaluation of their electrical properties by expressing them in *Xenopus* oocytes.

**Figure 7.**
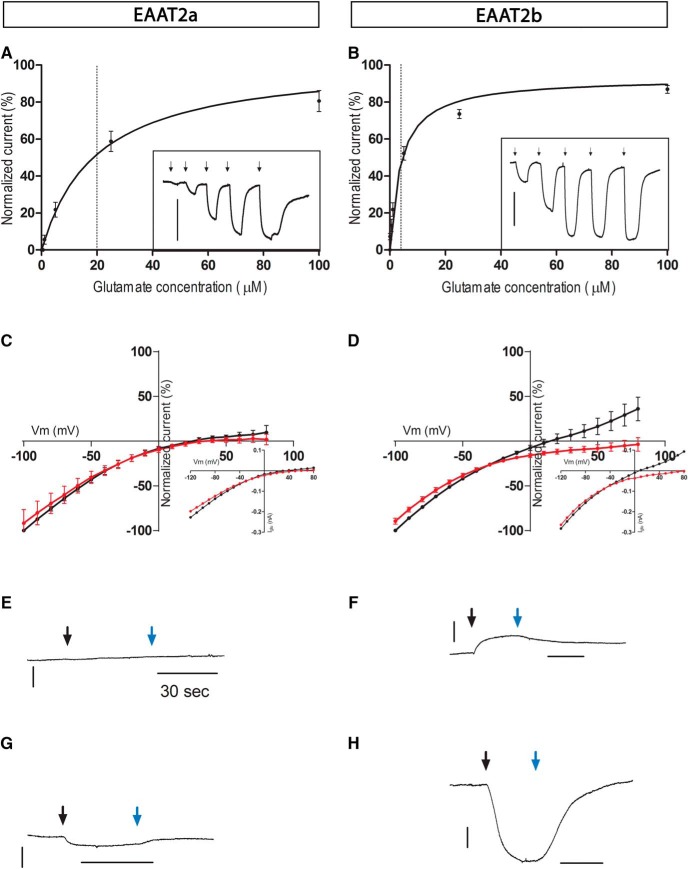
Two-electrode voltage clamp recordings from EAAT2a- and EAAT2b-expressing oocytes. ***A***, ***B***, Glutamate-evoked currents normalized to the saturating current induced by 500 µM glutamate in EAAT2a-expressing (***A***) and EAAT2b-expressing (***B***) oocytes (*n* = 8) and fitted with the Michaelis–Menten equation. The oocytes were voltage clamped at –50 mV. For clarity, fitted curves were plotted only up to 100 µM. In the insets, inward currents were induced by increasing concentrations of l-glutamate (1, 5, 25, 100, and 500 µM) in representative oocytes. The arrows indicate when glutamate was applied. Scale bar is 20 nA. ***C***, ***D***, Voltage dependence of EAAT2a-mediated (C) and EAAT2b-mediated (D) currents (*n* = 5) induced by 100 µM l-glutamate in control solution (black) and a chloride-free solution (red). The data from each cell were normalized to the response elicited by 100 µM l-glutamate in control solution at –100 mV. Insets show I-V recordings from representative oocytes in normal buffer. Data under both conditions are recorded from the same cells; oocytes are from three different batches. ***E–H***, TBOA reveals a leak current. TBOA (100 mM) was applied alone (black arrow) in control medium to EAAT2a (***E***) and EAAT2b (***F***) injected oocytes. In EAAT2b injected oocytes, it evoked an outward current. When oocytes were dialyzed with a chloride-free solution for 24 h and the control perfusing medium was exchanged with a solution containing SCN^-^ as the main negative ion, TBOA induced an inward current in EAAT2a and EAAT2b injected oocytes (***G***, ***H***). Blue arrows indicate wash from TBOA. Scale bar is 10 nA.

Approximately 48 h after microinjection of *eaat2a* or *eaat2b* mRNA, *Xenopus* oocytes displayed glutamate-evoked currents (I_Glu_) that were absent in uninjected oocytes. In oocytes voltage-clamped to negative potentials, increasing doses of glutamate evoked an increasing inward current (Fig. 7*A*, *B*, insets). The normalized amplitude of I_Glu_ for different glutamate concentrations was fitted with the Michaelis–Menten equation (Fig. 7*A*, *B*) to yield a *K_m_* of 19.6 ± 2.7 µm (*n* = 6) for EAAT2a and 3.1 ± 0.6 µm (*n* = 8) for EAAT2b (see Materials and Methods). When voltage-clamping the oocytes to different potentials and measuring I_Glu_ in the steady state, we found that at sufficiently positive potentials, I_Glu_ reversed for both EAAT2a- and EAAT2b-injected oocytes (Fig. 7*C*, *D*, black). This outward current was more prominent for EAAT2b-expressing oocytes. Such an outward current has been described for human EAAT1 and EAAT3 and salamander sEAAT1, sEAAT2, and sEAAT5 (Eliasof and Werblin, 1993; Wadiche et al., 1995; Eliasof et al., 1998a, b) and shown to be due to the uncoupled chloride conductance that is associated with the transporter itself.

The more prominent outward current in EAAT2b-injected oocytes suggested that a larger chloride conductance was associated with this paralog. To verify this hypothesis, we recorded I_Glu_ in a bath solution in which chloride was substituted with gluconate. Gluconate cannot permeate the chloride channel; therefore, for potentials that are positive to the chloride equilibrium potential, no current carried by an inward chloride flux should be recorded. For EAAT2a-injected oocytes, there was no difference between the glutamate current recorded in control solution and that recorded in chloride-free solution (Fig. 7*C*). Conversely, for EAAT2b-injected oocytes, the glutamate-induced outward current was abolished at positive potentials when recorded in a gluconate-based solution, whereas the inward current was only minimally affected (Fig. 7*D*). The chloride current is therefore a significant component of the total glutamate-induced current.

To estimate how much of the total I_Glu_ was due to the uncoupled chloride component, we calculated the charge-to-flux ratio (the charge transferred per molecule of glutamate taken up), using bath-applied tritiated glutamate. This ratio is independent of functional expression-level efficiency and provides a reliable measure of the current elicited by each molecule of glutamate. At a holding potential of –60 mV, the calculated charge-to-flux ratios were significantly different (*p* < 0.0008, two-tailed *t* test) for EAAT2a (1.7 ± 0.4, *n* = 8) and EAAT2b (4.5 ± 1.7, *n* = 8) injected oocytes.

However, at this potential, the recorded current is due to both the chloride conductance and the stoichiometrically transported ions (Na^+^, K^+^, and H^+^). To confirm that the difference between the two ratios was indeed due to chloride flux, we repeated the uptake experiment at –15 mV, the calculated chloride equilibrium potential (see Materials and Methods). The charge-to-flux ratio did not differ for the two transporters (1.2 ± 0.2 for EAAT2a and 1.3 ± 0.3 for EAAT2b) at this potential. Therefore, we concluded that the observed difference in charge-to-flux ratio at –60 mV was indeed due to differences in chloride conductance. Moreover, the charge-to-flux ratio at –60 mV for EAAT2a was not significantly different from the uptake at –15 mV (ANOVA plus Tukey *post hoc* test), which indicated that the larger charge-to-flux ratio measured at –60 mV with EAAT2b-injected oocytes was mostly due to uncoupled chloride current.

### EAAT2a and EAAT2b differ in their leak currents

To determine whether currents were induced only by the ligand glutamate, we checked whether DL-TBOA, a nontransportable antagonist, could block I_Glu_ in EAAT2a- and EAAT2b-injected oocytes. DL-TBOA blocked the glutamate-induced current as expected, but also revealed a current in EAAT2b (not in EAAT2a) injected oocytes in the absence of glutamate (Fig. 7*E*, *F*). Indeed, the antagonist blocks the channel, preventing the ions from flowing through it, and unmasks ion slippage through the transporter in the absence of substrate (glutamate in this case; Shimamoto et al., 2004).

The leak current (determined by subtracting the current in DL-TBOA from the current recorded in absence of antagonist) was inward at negative potentials and outward for potentials more positive than –16 mV (not shown). Leak currents have been described for most electrogenic carriers (Sonders and Amara, 1996; Ryan et al., 2004; Vandenberg et al., 2008; Andrini et al., 2008), but their function is often not clear. For some transporters, the ions contributing to the leak current can be the same ones that are translocated, whereas for others they are different. The calculated oocyte chloride equilibrium potential is close to the reversal potential of the EAAT2b leak current, suggesting that chloride flows through EAAT2b in the absence of glutamate. In contrast to EAAT2b, we never observed an outward current at negative potentials in the presence of DL-TBOA in EAAT2a-injected oocytes (*n* = 6).

To test whether the absence of a leak current in EAAT2a could be due to the absence of such ion slippage or to a negligible chloride conductance associated with the transporter, we recorded the DL-TBOA–induced currents in a solution in which chloride was substituted with SCN^-^. When DL-TBOA alone was applied to the SCN^-^ bathing solution, an inward current was observed in all EAAT2b-injected oocytes at negative potentials (Fig. 7*H*): in these experimental conditions, SCN^-^ was indeed flowing into the oocyte through the EAAT2b before DL-TBOA closed the leak. Under these same conditions, even EAAT2a oocytes displayed an inward current, although small compared with the one elicited in EAAT2b-injected oocytes (Fig. 7*G*). This current was too small to be measured in EAAT2a-expressing oocytes bathed in standard Ringer solution, suggesting that only EAAT2b has a leak current that could exert an important physiologic role.

## Discussion

The synaptic clearance of glutamate by EAATs is essential to maintain synaptic function in the CNS (Zhou and Danbolt, 2013). In this study, we focused on zebrafish EAAT2 proteins that are expressed at the photoreceptor synapse. This paralogous gene pair originated from a genome-duplication event ∼350 million years ago. Intriguingly, we found the two paralogous genes and their proteins to be complementarily expressed in two distinct retinal cell types: cone photoreceptors and Müller glia cells. Although high mRNA expression levels of *eaat2a* are observed in the INL, only low transcript levels can be seen in photoreceptors. On the other hand, mRNA expression levels of *eaat2b* are high in photoreceptors and low in the INL. This weak INL expression of *eaat2b* and even lower transcript levels of *eaat2a* in photoreceptors might be a remnant of the ancestral *eaat2* gene expression, which was, before the whole-genome duplication, likely expressed in both Müller glia cells and photoreceptors. Interestingly, protein expression of the corresponding *eaats* in these cells could not be detected using our immunofluorescence protocol. Whether this is due to the generally low protein concentration in the membrane of these cells or degradation of EAAT2b in Müller cells and EAAT2a in cones remains to be analyzed. The case of EAAT2 represents a rare case in which the subfunctionalization event led to a change not only in expression pattern but also in biophysical properties that can be directly linked to the biological function.

Unfortunately, morpholino antisense nucleotides are effective in knocking down protein only until ∼5 dpf. At this stage, the small size of the larval retina prevents patch-clamp recordings of outer and inner retinal neurons. Furthermore, no paralog-specific pharmacologic inhibitors are available that would allow examination of the transporters in the adult retina by single-cell recordings. Therefore, functional analysis was conducted by ERG recordings. The ERG measures sum field potential changes of the retina evoked by changes in illumination. It has proven to be a robust measure of photoreceptor responses and subsequent bipolar cell activation. The ERG is a standard approach to measure outer retina function not only in model organisms, but also in the clinic (Perlman, 1995).

**Figure 8.**
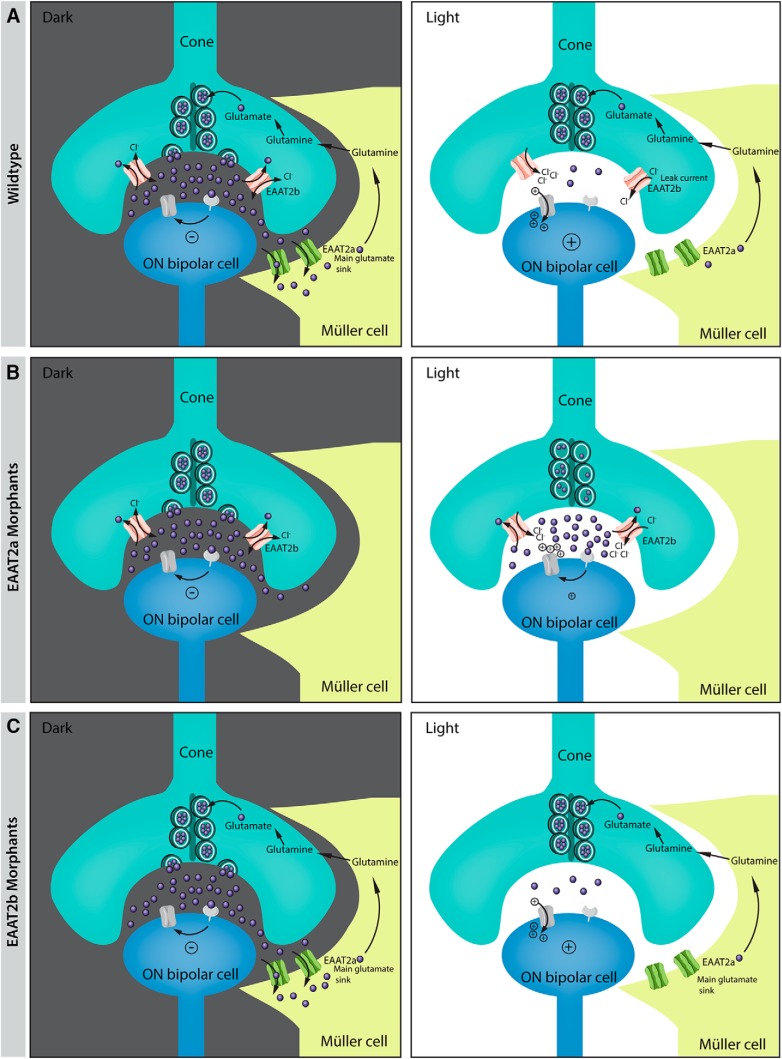
Schematics of photoreceptor synapse in light and dark. Illustration of changes in the photoreceptor synapse between light and dark in WT (***A***) and EAAT2a (***B***) and EAAT2b (***C***) morphants. EAAT2a on Müller glia cells is responsible for the uptake of the main load of glutamate. The uptaken glutamate in Müller cells is recycled via the glutamate–glutamine cycle. In EAAT2a morphants (***B***), the main load of glutamate is not being taken up and the cleft glutamate concentrations remain high, even during bright light stimuli. This may lead to binding of glutamate to postsynaptic receptors and therefore to a decreased ON-response in comparison to WT animals. The presynaptic transporter EAAT2b has a large Cl^-^ conductance with a large leak current in absence of glutamate. During a light stimulus, when photoreceptors hyperpolarize and very few glutamate remains in the synaptic cleft, Cl^-^ leaves the photoreceptor (leak current) and brings back the membrane potential closer to the dark resting potential. Because of the lack of such a leak current in EAAT2b morphants, cones remain in a slightly more hyperpolarized state.

Knockdown of the glial transporter EAAT2a shows a highly significant reduction in the ERG b-wave, implying that glutamate levels in the synaptic cleft remain elevated even during a light stimulus, saturating postsynaptic glutamatergic receptors (Fig. 8*B*). This is consistent with the increased time to peak recorded for the b-wave in these animals. This delay is likely due to elevated glutamate levels in the cleft, even after light stimulation. In darkness, photoreceptors tonically release glutamate, which binds to the postsynaptic metabotropic glutamate receptor 6b (Nomura et al., 1994; Huang et al., 2012). This leads, via a signaling cascade, to the closure of the cation-conducting ion channel TRPM1 (Morgans et al., 2009). During a light stimulus, photoreceptors become more hyperpolarized, which results in a decreased number of vesicles fusing at the presynapse. This, together with glutamate transporters clearing the synaptic glutamate, results in reduced concentrations of glutamate in the synaptic cleft, to an extent depending on the light stimulus intensity (Fig. 8*A*). In EAAT2a-depleted animals, glutamate levels do not decrease during a light stimulus to the same extent as in WT animals, implying reduced and delayed ON bipolar cell depolarization (Fig. 8*B*). Müller glia cell processes wrap around and coat photoreceptor synapses but do not invaginate the synapse (Burris et al., 2002). Thus released glutamate needs to diffuse from the presynaptic release site to the glial glutamate transporter EAAT2a on Müller cell processes, where it is being taken up. Such a system, with the main transporter being outside the synaptic cleft, prevents direct competition of transporter with the postsynaptic receptors and therefore keeps interference of the glutamate uptake with signal propagation to a minimum (Gaal et al., 1998; Roska et al., 1998). In accordance with being expressed on glia cells rather than neurons, we could not associate EAAT2a with high chloride conductance or with a high leak current, which implies that its primary function is high-capacity glutamate transport and not modulation of membrane potentials.

Loss of the presynaptic glutamate transporter EAAT2b only slightly modulates the ERG b-wave, indicating that synaptic glutamate levels in knockdown animals must still be comparable to the ones in WT (Fig. 8*C*). The fact that the kinetics of the EAAT2b morphant ERG does not significantly differ from that in control animals further reveals that EAAT2b does not have a high glutamate turnover rate. However, EAAT2b shows a high affinity to glutamate; hence, it must either be significantly outnumbered by EAAT2a or have a lower cycling rate or capture efficiency (likelihood of glutamate being transported versus released again) than EAAT2a. Glutamate transporters have been shown to buffer glutamate and thereby shape signal transmission after a quantal event in a fast time frame, despite having slow turnover rates (Wadiche et al., 1995; Diamond and Jahr, 1997; Wadiche and Kavanaugh, 1998). Furthermore, EAAT1–3 are thought to have a capture efficiency of 0.5, indicating that the likelihood of being released again equals the likelihood of being transported (Cavelier and Attwell, 2005; Beart and O’Shea, 2007; Tilleux and Hermans, 2007). Therefore, we assume that glutamate released by photoreceptors can bind and unbind several transporters within the cleft, before being taken up by glial transporters that surround the synapse in high numbers (Diamond and Jahr, 1997; Wadiche and Jahr, 2001; Bergles et al., 2002).

In addition, our ERG results suggest that the main vesicular glutamate pool of cones is not being replenished by presynaptic glutamate uptake via EAAT2b; otherwise EAAT2b knockdown would result in a depletion of the presynaptic vesicles, which would give us a more drastic phenotype. Glutamate vesicles of cones therefore seem to be mainly filled by glutamate recycled via the glutamate–glutamine cycle between Müller cells and photoreceptors (Fig. 8*A*), revealing another difference between cones and rods, which are thought to possess an almost self-sustaining glutamate uptake system (Hasegawa et al., 2006).

ERG recordings on double morphant zebrafish indicate that upon loss of both EAAT2 paralogs, glutamate uptake is further neutralized. The resulting difference in the b-wave amplitude between EAAT2a single loss and EAAT2 double knockdown is (1) the result of photoreceptors possessing a limited pool of glutamate-filled vesicles (as both the glutamate–glutamine cycle via Müller glia cells and the presynaptic uptake of glutamate are inhibited); (2) the result of an increased glutamate level in the synaptic cleft, even during light stimuli; or (3) both.

Binding of glutamate by EAAT2b is accompanied by the opening of chloride conductance that generates a large component of the total current. The chloride driving force and ionic flux direction depend on the chloride equilibrium potential that we suppose for zebrafish cones is between –40 and –50 mV. These estimates stem from data obtain in goldfish (–50 mV; Vroman et al., 2014), salamanders (–46 mV; Thoreson and Bryson, 2004), and turtle (–47 mV; Kaneko and Tachibana, 1986) cones. As the cone dark resting potential of zebrafish is around –40 mV (Endeman et al., 2013), very close to the chloride equilibrium potential (E_Cl-_), the flux of chloride that accompanies glutamate transport of EAAT2b will force the cone to return to potentials close to the dark resting potential, giving rise to previously described positive feedback (Szmajda and Devries, 2011). The presence of the large leak current that we have described for EAAT2b in the absence of glutamate will push the cone toward the resting potential, even when glutamate in the cleft is extremely low. Accordingly, a prolonged and strong light stimulus will speed up the repolarization at light off (Rowan et al., 2010), stabilizing the resting potential at depolarized potentials (Fig. 8*C*). The lack of such a chloride leak in EAAT2b knockdown larvae would maintain the cones in a slightly hyperpolarized status in darkness and therefore contribute to the observed slight b-wave reduction. This effect would become more evident in the double knockdown larvae. Indeed, in this case, the postsynaptic glutamatergic receptors would be partially saturated owing to the increased glutamate concentration in the cleft, and the hyperpolarization of the cones in darkness (and their possible loss of readily available vesicles) would further reduce the difference in cleft glutamate between dark and light conditions.

It is intriguing that the zebrafish retina acquired a system with subfunctionalized EAAT2 proteins that is comparable to the one present in the mouse retina with different EAATs. In contrast to zebrafish, mice use EAAT2 as the presynaptic transporter, whereas EAAT1 (GLAST) on Müller cells constitutes the main glutamate sink of the photoreceptor synapse (Tse et al., 2014).

## Synthesis

Reviewing Editor: Siegrid Löwel, University of Goettingen Decisions are customarily a result of the Reviewing Editor and the peer reviewers coming together and discussing their recommendations until a consensus is reached. When revisions are invited, a fact-based synthesis statement explaining their decision and outlining what is needed to prepare a revision will be listed below. The following reviewer(s) agreed to reveal their identity: Timm Schubert

The manuscript by Stephanie Niklaus et al. represents an interesting study on the localization and possible function of two glutamate transporters, EAAT2a and EAAT2b, at the cone synapse in the zebrafish retina. The authors provide histological evidence for a cell specific localization of the transporters, with the help of gene knockdown experiments and ERG recordings reveal possible functions of the transporters, and with patch clamp recordings analyzed transporter current properties in glutamate transporter expressing oocytes.

Both reviewers agreed that your study was solid, well performed and easy to read, but -regarding the level of the experimental analysis -it does not yet reach the high quality of a study to be published in eNeuro. The main reason for that conclusion is that your ERG recordings - while being a valid method to study the general physiological state of the retina - lack specificity with respect to your major topic, the role of the glutamate transporters for shaping signal transmission at the first synapse in retinal synaptic transmission. The correlative results from the ERG recordings thus do not allow a direct functional assignment of EAAT function at the cone synapse.

Both reviewers and I also agreed that the investigated topic is interesting enough for a broader neuroscience community and that you should have the chance to resubmit your work to eNeuro, after few additional experiments which should be doable within a few weeks.

We therefore would like to invite you to perform patch-clamp recordings of the involved cells:

1. from zebrafish cones (not only oocytes) since the EAAT function could be different in cones and oocytes (recordings of isolated cone containing the the axon terminal system or in the slice would be helpful), and

2. also to measure the effect of EAATa and b blockage in ON-BCs while stimulating with light in the retinal slice.

Minor points raised by the reviewers:

1. In line 282-283 the authors write “We did not observe any histological changes of the retina, in EAAT2a or EAAT2b morphants (Figure 4)". This statement should be supported by a quantitative morphological analysis. For example, it would be informative whether the morphology of the Müller cells and/or cone photoreceptors shows any alterations.

3. Close inspection of Figure 1 shows that eaat2a mRNA is weakly expressed in the PhR layer in addition to the INL. Also eaat2b mRNA is present in both retinal layers. These findings suggest that photoreceptors and Müller cells express a mixture of both transporters. This should have an effect on the ERG, something that is not satisfactorily described and discussed in the paper.

4. In Figure 5D-F one cannot see the start of the light stimulus.

5. In Figure 5F the time to peak value (if the stimulus ends at time 0) in the log-4 ERG trace looks to be around 230ms but in the quantification (Figure 6), the value is around 370ms?

6. In Figure 5E, the ERG recording shows first a negative then a positive peak. Could it be that the first negative peak (where the b-wave is located in the control ERG) is in fact the b-wave and the positive peak is a later component of the ERG? Actually, this deflection preceding the b-wave cannot be observed in D and F, and you should offer an explanation for it.

7. It would help the reader if the authors would describe in more detail their ideas how variations of glutamate concentration in the synaptic cleft affect the amplitude and timing of the b-wave when stepping from dark to light adapted conditions.

8. Page 11, line 270: ‘...we used transgenic fish lines expressing GFP in different photoreceptor cell types...’, please consider to name these lines rather than just giving the references.

9. Page 4, line 77/78: ‘....the release of thousands of vesicles....’ Really?, thousands seems to be high, hundreds would be more reasonable.

10. Page 4, lines 92-95: ‘....EAAT2 is expressed...is located presynaptically in cone pedicles.’ These are statements that are essential for this manuscript. Please give references for these statements

11. Page 6, line 142: please give the reader the information what zpr1 stands for.

12. P10, line 236: Amphoterin B was used in the electrode for perforated patch. Please give the reader the information in the Methods that the perforated patch recording were used.

13. P10, line 246: remove ‘mV’ after -45.5

14. Figure 1: vertical labeling, add space between 5 and dpf. In A but also in C there is another labelled structure above the INL (and under the lense). Consider stating what it is to avoid confusion that EAAT could be expressed in the GCL, maybe in the legends.

15. Figures 3 to 5: Consider a space between the number and ng, add scale bars. Also, the authors could give the concentration in brackets after ‘EEAT2a/b MO 2’

## References

[B1] Andrini O, Ghezzi C, Murer H, Forster IC (2008) The leak mode of type II Na(+)-P(i) cotransporters. Channels (Austin, Tex.) 2:346–357. 18989094 10.4161/chan.2.5.6900

[B2] Barnett NL, Pow DV (2000) Antisense knockdown of GLAST, a glial glutamate transporter, compromises retinal function. Invest Ophthalmol Vis Sci 41:585–591. 10670492

[B3] Beart PM, O’Shea RD (2007) Transporters for L-glutamate: an update on their molecular pharmacology and pathological involvement. Br J Pharmacol 150:5–17. 10.1038/sj.bjp.0706949 17088867 PMC2013845

[B4] Bergles DE, Tzingounis AV, Jahr CE (2002) Comparison of coupled and uncoupled currents during glutamate uptake by GLT-1 transporters. J Neurosci 22:10153–10162. 12451116 10.1523/JNEUROSCI.22-23-10153.2002 PMC6758767

[B5] Branchek T (1984) The development of photoreceptors in the zebrafish, brachydanio rerio. II. Function. J Comp Neur 224:116–122. 10.1002/cne.902240110 6715575

[B6] Branchek T, Bremiller R (1984) The development of photoreceptors in the zebrafish, Brachydanio rerio. I. Structure. J Comp Neur 224:107–115. 10.1002/cne.902240109 6715574

[B7] Burris C, Klug K, Ngo IT, Sterling P, Schein S (2002) How Müller glial cells in macaque fovea coat and isolate the synaptic terminals of cone photoreceptors. J Comp Neur 453:100–111. 10.1002/cne.10397 12357435

[B8] Cavelier P, Attwell D (2005) Tonic release of glutamate by a DIDS-sensitive mechanism in rat hippocampal slices. J Physiol 564:397–410. 10.1113/jphysiol.2004.082131 15695241 PMC1464434

[B9] Diamond JS, Jahr CE (1997) Transporters buffer synaptically released glutamate on a submillisecond time scale. J Neurosci 17:4672–4687. 9169528 10.1523/JNEUROSCI.17-12-04672.1997 PMC6573331

[B10] Eliasof S, Arriza JL, Leighton BH, Amara SG, Kavanaugh MP (1998a) Localization and function of five glutamate transporters cloned from the salamander retina. Vis Res 38:1443–1454. 9667010 10.1016/s0042-6989(97)00452-5

[B11] Eliasof S, Arriza JL, Leighton BH, Kavanaugh MP, Amara SG (1998b) Excitatory amino acid transporters of the salamander retina: identification, localization, and function. J Neurosci 18:698–712. 9425012 10.1523/JNEUROSCI.18-02-00698.1998 PMC6792528

[B12] Eliasof S, Werblin F (1993) Characterization of the glutamate transporter in retinal cones of the tiger salamander. J Neurosci 13:402–411. 8093715 10.1523/JNEUROSCI.13-01-00402.1993 PMC6576323

[B13] Endeman D, Klaassen LJ, Kamermans M (2013) Action spectra of zebrafish cone photoreceptors. PloS One 8:e68540. 10.1371/journal.pone.0068540 23861916 PMC3702584

[B14] Fairman WA, Vandenberg RJ, Arriza JL, Kavanaugh MP, Amara SG (1995) An excitatory amino-acid transporter with properties of a ligand-gated chloride channel. Nature 375:599–603. 10.1038/375599a0 7791878

[B15] Gaal L, Roska B, Picaud SA, Wu SM, Marc R, Werblin FS (1998) Postsynaptic response kinetics are controlled by a glutamate transporter at cone photoreceptors. J Neurophysiol 79:190–196. 9425190 10.1152/jn.1998.79.1.190

[B16] Gesemann M, Lesslauer A, Maurer CM, Schönthaler HB, Neuhauss SC (2010a) Phylogenetic analysis of the vertebrate excitatory/neutral amino acid transporter (SLC1/EAAT) family reveals lineage specific subfamilies. BMC Evol Biol 10:117. 20429920 10.1186/1471-2148-10-117 PMC2873418

[B17] Gesemann M, Maurer CM, Neuhauss SC, Stephan CF (2010b) Excitatory amino acid transporters in the zebrafish: Letter to “Expression and functional analysis of Na(+)-dependent glutamate transporters from zebrafish brain” from Rico et al., Brain Res Bull 83:202–206. 20466040 10.1016/j.brainresbull.2010.04.018

[B18] Glasauer SS, Neuhauss SC (2014) Whole-genome duplication in teleost fishes and its evolutionary consequences. Mol Genet Genom 289:1045–1060. 10.1007/s00438-014-0889-2 25092473

[B19] Hamaoka T, Takechi M, Chinen A, Nishiwaki Y, Kawamura S (2002) Visualization of rod photoreceptor development using GFP-transgenic zebrafish. Genesis (New York) 34:215–220. 10.1002/gene.10155 12395387

[B20] Harada T, Harada C, Watanabe M, Inoue Y, Sakagawa T, Nakayama N, Sasaki S, Okuyama S, Watase K, Wada K, Tanaka K (1998) Functions of the two glutamate transporters GLAST and GLT-1 in the retina. Proc Natl Acad Sci U S A 95:4663–4666. 9539795 10.1073/pnas.95.8.4663 PMC22547

[B21] Hasegawa J, Obara T, Tanaka K, Tachibana M (2006) High-density presynaptic transporters are required for glutamate removal from the first visual synapse. Neuron 50:63–74. 10.1016/j.neuron.2006.02.022 16600856

[B22] Huang Y-Y, Haug MF, Gesemann M, Neuhauss SC (2012) Novel expression patterns of metabotropic glutamate receptor 6 in the zebrafish nervous system. PloS One 7:e35256. 10.1371/journal.pone.0035256 22523578 PMC3327648

[B23] Kaneko A, Tachibana M (1986) Effects of gamma-aminobutyric acid on isolated cone photoreceptors of the turtle retina. J Physiol 373:443–461. 3746679 10.1113/jphysiol.1986.sp016057 PMC1182547

[B24] Larison KD, Bremiller R (1990) Early onset of phenotype and cell patterning in the embryonic zebrafish retina. Development (Cambridge, England) 109:567–576. 10.1242/dev.109.3.567 2401210

[B25] Levinger E, Zemel E, Perlman I (2012) The effects of excitatory amino acids and their transporters on function and structure of the distal retina in albino rabbits. Doc Ophthalmol 125:249–265. 10.1007/s10633-012-9354-x 23054160

[B26] Levy LM, Warr O, Attwell D (1998) Stoichiometry of the glial glutamate transporter GLT-1 expressed inducibly in a Chinese hamster ovary cell line selected for low endogenous Na+-dependent glutamate uptake. J Neurosci 18:9620–9628. 9822723 10.1523/JNEUROSCI.18-23-09620.1998 PMC6793325

[B27] Makhankov YV, Rinner O, Neuhauss SC (2004) An inexpensive device for non-invasive electroretinography in small aquatic vertebrates. J Neurosci Methods 135:205–210. 10.1016/j.jneumeth.2003.12.015 15020104

[B28] McKeown KA, Moreno R, Hall VL, Ribera AB, Downes GB (2012) Disruption of Eaat2b, a glutamate transporter, results in abnormal motor behaviors in developing zebrafish. Dev Biol 362:162–171. 10.1016/j.ydbio.2011.11.001 22094018 PMC4013685

[B29] Morgans CW (2000) Neurotransmitter release at ribbon synapses in the retina. Immunol Cell Biol 78:442–446. 10.1046/j.1440-1711.2000.00923.x 10947871

[B30] Morgans CW, Zhang J, Jeffrey BG, Nelson SM, Burke NS, Duvoisin RM, Brown RL (2009) TRPM1 is required for the depolarizing light response in retinal ON-bipolar cells. Proc Natl Acad Sci U S A 106:19174–19178. 10.1073/pnas.0908711106 19861548 PMC2776419

[B31] Mullins MC, Hammerschmidt M, Haffter P, Nüsslein-Volhard C (1994) Large-scale mutagenesis in the zebrafish: in search of genes controlling development in a vertebrate. Curr Biol 4:189–202. 10.1016/S0960-9822(00)00048-8 7922324

[B32] Nomura A, Shigemoto R, Nakamura Y, Okamoto N, Mizuno N, Nakanishi S (1994) Developmentally regulated postsynaptic localization of a metabotropic glutamate receptor in rat rod bipolar cells. Cell 77:361–369. 8181056 10.1016/0092-8674(94)90151-1

[B33] Owe SG, Marcaggi P, Attwell D (2006) The ionic stoichiometry of the GLAST glutamate transporter in salamander retinal glia. J Physiol 577:591–599. 10.1113/jphysiol.2006.116830 17008380 PMC1890427

[B34] Palmer MJ, Taschenberger H, Hull C, Tremere L, von Gersdorff H (2003) Synaptic activation of presynaptic glutamate transporter currents in nerve terminals. J Neurosci 23:4831–4841. 12832505 10.1523/JNEUROSCI.23-12-04831.2003 PMC3586552

[B35] Perlman I (1995) The Electroretinogram: ERG In: Webvision: The Organization of the Retina and Visual System (Kolb H, Fernandez E, Nelson R, eds). University of Utah, Salt Lake City, UT. 21413389

[B36] Picaud SA, Larsson HP, Grant GB, Lecar H, Werblin FS (1995) Glutamate-gated chloride channel with glutamate-transporter-like properties in cone photoreceptors of the tiger salamander. J Neurophysiol 74:1760–1771. 8989410 10.1152/jn.1995.74.4.1760

[B37] Rauen T (2000) Diversity of glutamate transporter expression and function in the mammalian retina. Amino Acids 19:53–62. 11026473 10.1007/s007260070033

[B38] Rauen T, Taylor WR, Kuhlbrodt K, Wiessner M (1998) High-affinity glutamate transporters in the rat retina: a major role of the glial glutamate transporter GLAST-1 in transmitter clearance. Cell Tissue Res 291:19–31. 10.1007/s004410050976 9394040

[B39] Roska B, Gaal L, Werblin FS (1998) Voltage-dependent uptake is a major determinant of glutamate concentration at the cone synapse: an analytical study. J Neurophysiol 80:1951–1960. 9772252 10.1152/jn.1998.80.4.1951

[B40] Rowan MJ, Ripps H, Shen W (2010) Fast glutamate uptake via EAAT2 shapes the cone-mediated light offset response in bipolar cells. J Physiol 588:3943–3956. 10.1113/jphysiol.2010.191437 20807794 PMC3000584

[B41] Ryan RM, Mitrovic AD, Vandenberg RJ (2004) The chloride permeation pathway of a glutamate transporter and its proximity to the glutamate translocation pathway. J Biol Chem 279:20742–20751. 10.1074/jbc.M304433200 14982939

[B42] Schmitz F (2009) The making of synaptic ribbons: how they are built and what they do. Neurosci 15:611–624. 10.1177/1073858409340253 19700740

[B43] Shimamoto K, Sakai R, Takaoka K, Yumoto N, Nakajima T, Amara SG, Shigeri Y (2004) Characterization of novel L-threo-beta-benzyloxyaspartate derivatives, potent blockers of the glutamate transporters. Mol Pharmacol 65:1008–1015. 10.1124/mol.65.4.1008 15044631

[B44] Sonders MS, Amara SG (1996) Channels in transporters. Curr Opin Neurobiol 6:294–302. 8794089 10.1016/s0959-4388(96)80111-5

[B45] Szmajda BA, Devries SH (2011) Glutamate spillover between mammalian cone photoreceptors. J Neurosci 31:13431–13441. 10.1523/JNEUROSCI.2105-11.2011 21940436 PMC3212989

[B46] Takechi M, Hamaoka T, Kawamura S (2003) Fluorescence visualization of ultraviolet-sensitive cone photoreceptor development in living zebrafish. FEBS Lett 553:90–94. 14550552 10.1016/s0014-5793(03)00977-3

[B47] Takechi M, Seno S, Kawamura S (2008) Identification of cis-acting elements repressing blue opsin expression in zebrafish UV cones and pineal cells. J Biol Chem 283:31625–31632. 10.1074/jbc.M806226200 18796431

[B48] Thoreson WB, Bryson EJ (2004) Chloride equilibrium potential in salamander cones. BMC Neurosci 5:53. 10.1186/1471-2202-5-53 15579212 PMC539262

[B49] Tilleux S, Hermans E (2007) Neuroinflammation and regulation of glial glutamate uptake in neurological disorders. J Neurosci Res 85:2059–2070. 10.1002/jnr.21325 17497670

[B50] Tse DY, Chung I, Wu SM (2014) Pharmacological inhibitions of glutamate transporters EAAT1 and EAAT2 compromise glutamate transport in photoreceptor to ON-bipolar cell synapses. Vis Res 103:49–62. 10.1016/j.visres.2014.07.020 25152321 PMC4547049

[B51] Vandenberg RJ, Huang S, Ryan RM (2008) Slips, leaks and channels in glutamate transporters. Channels (Austin) 2:51–58. 10.4161/chan.2.1.6047 18690049

[B52] Vandenbranden CA, Verweij J, Kamermans M, Müller LJ, Ruijter JM, Vrensen GF, Spekreijse H (1996) Clearance of neurotransmitter from the cone synaptic cleft in goldfish retina. Vis Res 36:3859–3874. 9068839 10.1016/s0042-6989(96)00134-4

[B53] Veruki ML, Mørkve SH, Hartveit E (2006) Activation of a presynaptic glutamate transporter regulates synaptic transmission through electrical signaling. Nat Neurosci 9:1388–1396. 10.1038/nn1793 17041592

[B54] Vroman R, Klaassen LJ, Howlett MHC, Cenedese V, Klooster J, Sjoerdsma T, Kamermans M (2014) Extracellular ATP hydrolysis inhibits synaptic transmission by increasing pH buffering in the synaptic cleft. PLoS Biol 12:e1001864. 10.1371/journal.pbio.1001864 24844296 PMC4028192

[B55] Wadiche JI, Amara SG, Kavanaugh MP (1995) Ion fluxes associated with excitatory amino acid transport. Neuron 15:721–728. 7546750 10.1016/0896-6273(95)90159-0

[B56] Wadiche JI, Jahr CE (2001) Multivesicular release at climbing fiber-Purkinje cell synapses. Neuron 32:301–313. 11683999 10.1016/s0896-6273(01)00488-3

[B57] Wadiche JI, Kavanaugh MP (1998) Macroscopic and microscopic properties of a cloned glutamate transporter/chloride channel. J Neurosci 18:7650–7661. 9742136 10.1523/JNEUROSCI.18-19-07650.1998 PMC6793006

[B58] Wersinger E, Schwab Y, Sahel J-A, Rendon A, Pow DV, Picaud S, Roux MJ (2006) The glutamate transporter EAAT5 works as a presynaptic receptor in mouse rod bipolar cells. J Physiol 577:221–234. 10.1113/jphysiol.2006.118281 16973698 PMC2000664

[B59] White RD, Neal MJ (1976) The uptake of L-glutamate by the retina. Brain Res 111:79–93. 182318 10.1016/0006-8993(76)91050-7

[B60] Zerangue N, Kavanaugh MP (1996) Flux coupling in a neuronal glutamate transporter. Nature 383:634–637. 10.1038/383634a0 8857541

[B61] Zhou Y, Danbolt NC (2013) GABA and glutamate transporters in brain. Front Endocrinol 4:165. 10.3389/fendo.2013.00165 24273530 PMC3822327

